# CRISPR-FOCUS: A web server for designing focused CRISPR screening experiments

**DOI:** 10.1371/journal.pone.0184281

**Published:** 2017-09-05

**Authors:** Qingyi Cao, Jian Ma, Chen-Hao Chen, Han Xu, Zhi Chen, Wei Li, X. Shirley Liu

**Affiliations:** 1 State Key Laboratory for Diagnosis and Treatment of Infectious Diseases, Collaborative Innovation Center for Diagnosis and Treatment of Infectious Diseases, The First Affiliated Hospital, College of Medicine, Zhejiang University, Hangzhou, Zhejiang, P. R. China; 2 Department of Bioinformatics, School of Life Science and Technology, Tongji University, Shanghai, P. R. China; 3 Center for Functional Cancer Epigenetics, Dana-Farber Cancer Institute, Boston, MA, United States of America; 4 Department of Biostatistics and Computational Biology, Dana-Farber Cancer Institute and Harvard T.H. Chan School of Public Health, Boston, MA, United States of America; University of Arizona, UNITED STATES

## Abstract

The recently developed CRISPR screen technology, based on the CRISPR/Cas9 genome editing system, enables genome-wide interrogation of gene functions in an efficient and cost-effective manner. Although many computational algorithms and web servers have been developed to design single-guide RNAs (sgRNAs) with high specificity and efficiency, algorithms specifically designed for conducting CRISPR screens are still lacking. Here we present CRISPR-FOCUS, a web-based platform to search and prioritize sgRNAs for CRISPR screen experiments. With official gene symbols or RefSeq IDs as the only mandatory input, CRISPR-FOCUS filters and prioritizes sgRNAs based on multiple criteria, including efficiency, specificity, sequence conservation, isoform structure, as well as genomic variations including Single Nucleotide Polymorphisms and cancer somatic mutations. CRISPR-FOCUS also provides pre-defined positive and negative control sgRNAs, as well as other necessary sequences in the construct (e.g., U6 promoters to drive sgRNA transcription and RNA scaffolds of the CRISPR/Cas9). These features allow users to synthesize oligonucleotides directly based on the output of CRISPR-FOCUS. Overall, CRISPR-FOCUS provides a rational and high-throughput approach for sgRNA library design that enables users to efficiently conduct a focused screen experiment targeting up to thousands of genes.

(CRISPR-FOCUS is freely available at http://cistrome.org/crispr-focus/)

## Introduction

The Clustered Regularly Interspaced Short Palindromic Repeats (CRISPR)–CRISPR-associated system genes 9 (Cas9) system has been proving itself to be a prominent genome-editing technique [1–2]. Based on the CRISPR/Cas9 system, CRISPR screening is a high-throughput technology that enables researchers to examine the effect of perturbing tens of thousands of genes in parallel [3–5]. In a CRISPR-based screening experiment, single-guide RNA (sgRNA) pools designated to target different genomic loci are delivered into the cells by the lentivirus system, while the function of a gene can be inferred by comparing the abundance of cell populations bearing sgRNAs that target this particular gene across different conditions. CRISPR screening has been applied to interrogate gene functions in different contexts, including immune response [6–7], cancer progression [8–10] and metastasis [11], while recently this technique was being used to identify the functions of non-coding elements as well [12–18].

Many CRISPR screening experiments are conducted as unbiased, genome-scale approaches, where several genome-wide screening libraries are available [3,8–9,19]. On the other hand, focused screen is also conducted in many studies, where researchers use a small-scale library to target gene sets of specific interests (e.g., oncogenes/tumor suppressors for oncologists or cytokines for immunologists) [20], to validate hits of genome-wide screens [7], or to reduce the cost of screens (*e*.*g*., in *in vivo* settings [11]).

To design libraries for CRISPR screens (especially focused screens), several computational tools can be applied [19,21–30]. However, most of these algorithms provide optimized sgRNAs for only one or several genes/sequences [22–23,29]. A few web-based tools with nominal batch design capacity require users to provide target sequence for each individual gene, have strict size limits on the sequence file uploaded, could only accept limited numbers (10–20 mostly) of gene IDs as input, or base their work on mining of public domain libraries [19,25–26,30]. Some other tools with substantial batch-design capacity are not web-based, and require users to download the whole database, compile the source code and fine tune up to dozens of parameters [21,24,27–28]. Therefore, a user-friendly automatic tool is needed to facilitate the design process of CRISPR screen experiments.

Another issue of library design comes from the rational sgRNA evaluation and selection based on multiple criteria. Preferably, sgRNA should have fewer off-target effects (based on the alignment of spacer sequence across the whole genome [23,26–28]), and higher on-target knockout efficiencies (determined mainly by the sgRNA sequence context [19,31]), while it is proved necessary to consider both of them [9,32]. Other factors, like sequence conservation [20] and isoform structures of target genes [25,32], also have a marked impact on the results of the screen experiments. Once multiple scores are calculated for all candidate sgRNAs, a method will become necessary for sgRNAs prioritizing and filtering. Common practices include weight-averaging all scores by assigning a fixed (or empirical) weight for each criterion [19,24]; or applying the filters one by one, followed by ranking the candidates lexicographically [21]. These approaches might be too loose or too rigid in sgRNA selection, because the distribution of these scores might vary among different genes. To reach optimal sgRNA ranking results, an ideal method should consider all criteria, and summarize them appropriately in a context dependent way.

In light of requirements from CRISPR screen experiments, we developed CRISPR-FOCUS, a web-based method for library design of CRISPR screens. With minimum user input, CRISPR-FOCUS selects different numbers of sgRNAs targeting up to one thousand genes in human or mouse genome. SgRNAs in the output are ranked by their summary score, which is a comprehensive evaluation of efficiency, specificity, as well as target sequence conservation and the target of multiple isoforms. To our knowledge, CRISPR-FOCUS is the only web-based tool that is specially optimized for CRISPR screening experiments.

## Methods and implementation

### Overview

**Fig 1:**
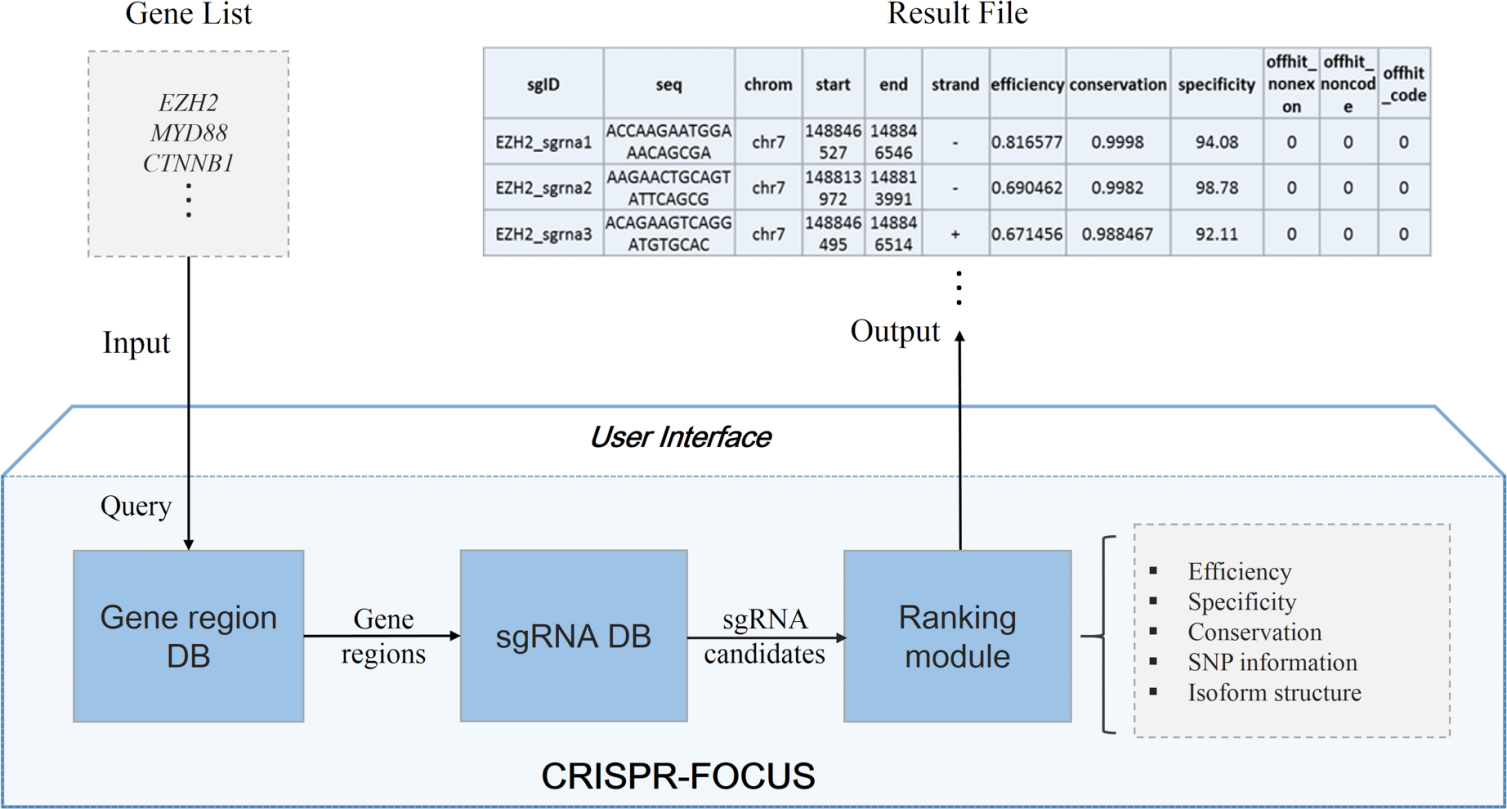
The main scheme of CRISPR-FOCUS.

The scheme of CRISPR-FOCUS is presented in Fig 1. All possible sgRNA candidates that have canonical Protospacer Adjacent Motif (PAM) in human and mouse genome are discovered and stored in the backend database. For each of the candidate sequence, all their attributes (described in details below) are pre-computed and stored. When user performs a query through the web interface, CRISPR-FOCUS will retrieve all possible candidates, prioritize them and return the top ones with highest scores.

### Criteria for sgRNA performance evaluation

To reach the best CRISPR-based knockout effect, the selection of sgRNAs should be optimized to (1) maximize their on-target cleavage effects (*i*.*e*., maximize efficiency), (2) minimize potential off-target effects (*i*.*e*., maximize specificity), (3) ensure the fidelity of their sequence with corresponding target loci (and to avoid regions with possible genomic variations), and (4) consider the importance of target region (evaluated by sequence conservation and isoform structure). CRISPR-FOCUS evaluates every sgRNA with the following indices.

#### Efficiency

The cleavage efficiency of a sgRNA is a major factor that determines the sensitivity of a screen experiment [4]. We used SSC [31], a computational algorithm that we previously developed to predict the cleavage efficiency of candidate sgRNAs. SSC takes spacer sequences as well as its flanking sequences as input, and uses Least Absolute Shrinkage and Selection Operator (LASSO) model to calculate an efficiency score for each sgRNA. CRISPR-FOCUS will filter sgRNAs with efficiency score below zero.

#### Specificity

For each candidate sgRNA, CRISPR-FOCUS first calculated its specificity score [33] to evaluate the overall similarity with putative off-target genomic loci. For sgRNAs that have perfect-match off-targets, we further divided them into three categories according to their off-target positions: (1) non-exon hits that do not overlap with exons of any coding or non-coding genes, (2) exon (but non-coding) hits that overlap with exons of non-coding genes, and (3) coding region hits that overlap with exons of coding genes. These sgRNAs may be considered in a rescue step (described later).

#### The effect of possible variations

SgRNAs are usually designed based on the reference genome sequence. The knockout efficiencies of these sgRNAs may be affected by the genomic sequences in cells that are different from the reference, especially mutation. CRISPR-FOCUS prefers sgRNAs that cover no or fewer mutations, including Single Nucleotide Polymorphisms (SNPs) and somatic mutations (especially in cancer). CRISPR-FOCUS retrieved SNP information from dbSNP [34], and annotated each sgRNA with all possible SNPs whose minor allele frequency (MAF) is higher than 0.05. sgRNAs that cover no or fewer variations will be preferentially chosen in the selection procedure. If screen experiments are conducted in cancer cells, users could also choose whether to avoid recurrent somatic mutations from different cancer types (using the COSMIC database [35]).

#### Sequence conservation

Regions in a gene with higher conservation rates across species are more likely to be important, as they usually encode conserved functional domains (like catalytic center for enzyme or DNA binding domain for transcriptional factor) whose knockout are more likely to disrupt gene function [20]. CRISPR-FOCUS annotated each sgRNA with an average phastCon conservation score [36] of the corresponding target position.

#### Isoform structure

Some genes have multiple isoforms (or transcripts) with different structures. To completely knockout a gene, a sgRNA should ideally target as many isoforms as possible. For each exon region, CRISPR-FOCUS calculates an “isoform commonality score”, which is defined as the percentage of isoforms that uses this exon. SgRNAs targeting exon regions with higher scores are preferred.

### SgRNA selection and ranking

For each gene in the query, CRISPR-FOCUS first retrieves all genomic coordinates of all exons, and collects all sgRNA candidates that overlap with these regions. It will next perform a “filter and rescue” procedure (described in S1 File in detail) to rank all candidates and pick up the top ones. For the filtering step, CRISPR-FOCUS will filter sgRNAs that are empirically regarded as “bad” candidates, including sgRNAs that: (1) overlap with a SNP or mutation loci, (2) contains >40% guanine counts (‘G’s), which is observed to have higher off-target effects [37], or (3) are perfectly matched to putative off-target loci within the genome. The remaining ones will be ranked by a summary score, which is a weighted summary of efficiency, specificity, phastCon conservation and exon commonality score, while all the weights are dynamically defined by the Criteria Importance Through Intercriteria Correlation (CRITIC) method [38]. The purpose of this method is to determine the objective weight for each criterion in multiple criteria decision problems. Briefly in CRITIC, a value *C*_*j*_ is calculated to quantify the amount of information transmitted by criterion *j*, which is determined by both contrast intensity and conflict of the decision criteria. The contrast intensity is represented by the standard deviation of *j*, while the conflict is measured as the multiplicative aggregation of one minus correlation coefficients between *j* and the rest of criteria. Finally, object weight *w*_*j*_ is generated by normalizing *C*_*j*_ to the unity of all *C* values.

**Fig 2:**
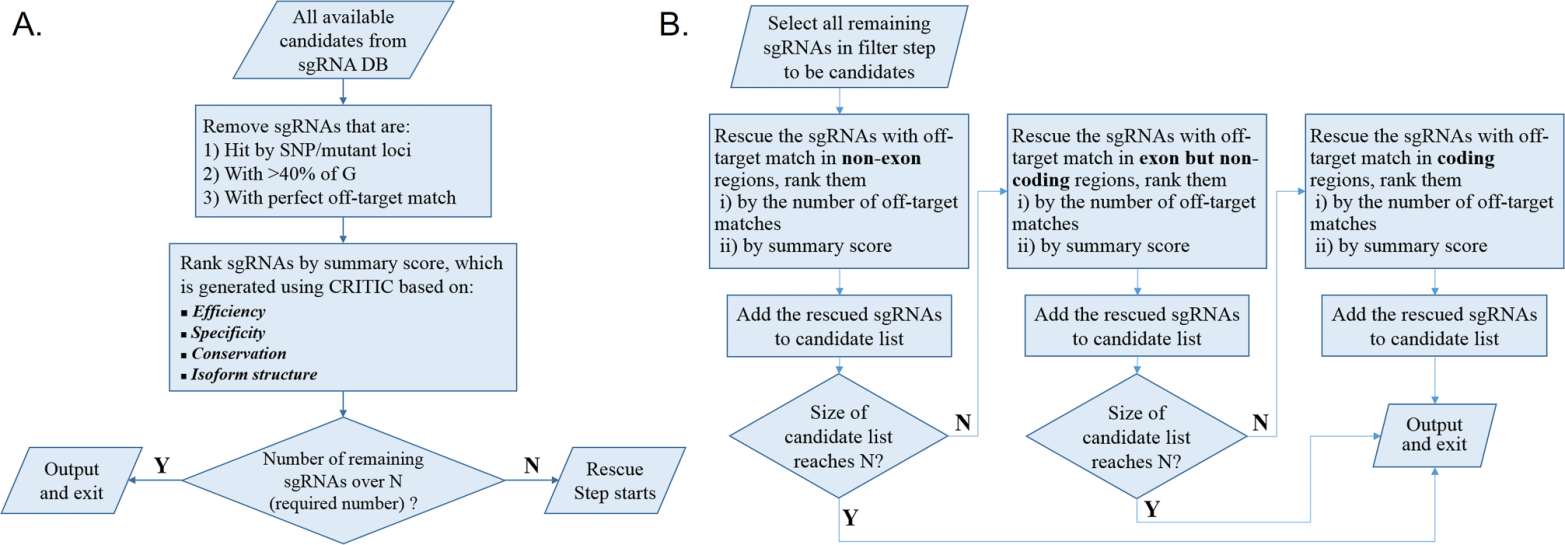
Workflow of the sgRNA selection/ranking process in CRISPR-FOCUS. The sgRNA selection/ranking process in CRISPR-FOCUS is composed of (A) a filter step and (B) a rescue step.

If the number of remaining sgRNAs does not reach the desired number, CRISPR-FOCUS will execute a “rescue” step to retrieve more possible sgRNAs. At this stage, sgRNAs with potential off-target hits will be rescued in the following order: (1) sgRNAs with non-exon off-target hits only, (2) sgRNAs with off-target hits located on non-coding elements but not coding regions, (3) sgRNAs with off-target hits located on coding regions. sgRNAs within the same category will be prioritized based on their number of off-target hits, or by the summary score if two candidates have the same number of hits within the same category. A detailed flowchart of the whole procedure is depicted in Fig 2.

### The web portal

**Fig 3:**
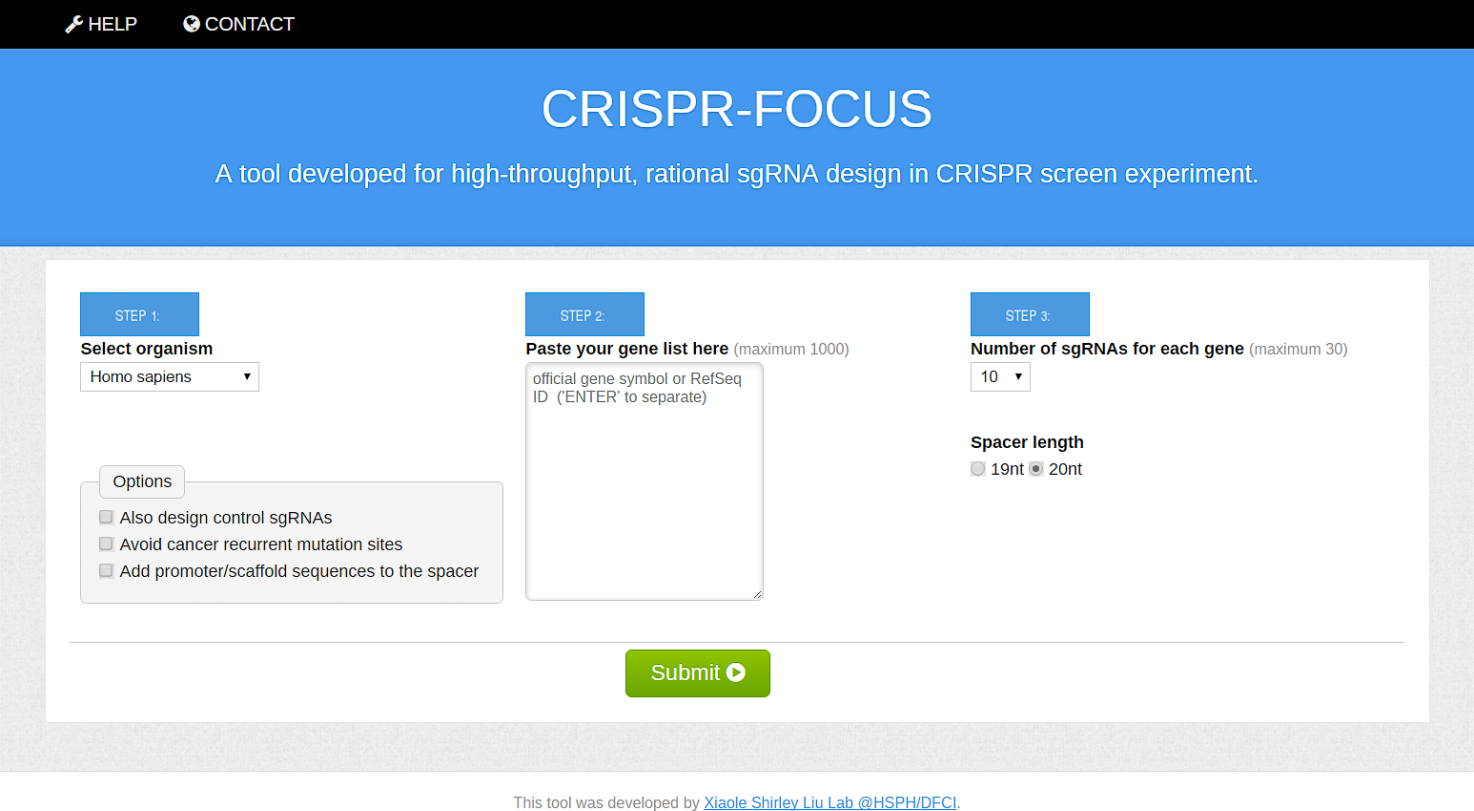
The main user interface of CRISPR-FOCUS. A screenshot of the CRISPR-FOCUS website (http://cistrome.org/crispr-focus/)) is shown.

The web portal of CRISPR-FOCUS (Fig 3) accepts a gene ID (either official gene symbol or RefSeq ID) list as input, and returns the designated number of sgRNA candidates per each gene. Users can input up to 1000 genes, and retrieve up to 30 sgRNAs per gene. Users can also select sgRNAs from either *Homo sapiens* or *Mus musculus*. The web portal applies Common Gateway Interface (CGI) to fetch input, while all backend scripts were written in Python programming language.

CRISPR-FOCUS also provides other options to accommodate different requirements, including the selection of different sgRNA lengths (19 or 20nt) [5,39]. As commonly used constituents of CRISPR/Cas9 delivery system, human U6 promoter and spCas9 scaffold could be appended to the output, allowing users to synthesize the library directly from the output. Furthermore, CRISPR-FOCUS includes a set of negative control sgRNAs (targeting several known “safe-harbor” loci within human or mouse genome) [40–41] and positive control sgRNAs (targeting 58 essential ribosome genes identified in [31]). The input and output formats are described in Table A in S2 File. The execution of CRISPR-FOCUS is based on genome assembly hg38 (for human) and mm10 (for mouse), while full versions of public domain databases applied to annotate sgRNAs could be found in Table B in S2 File.

## Results and discussion

CRISPR-FOCUS provides a high throughput platform for rational sgRNA library design of CRISPR screen experiment. It could accomplish a full scale design (up to 1000 target genes with 30 sgRNAs for each) within about twenty seconds. To our knowledge, CRISPR-FOCUS is now the only web-based sgRNA design tool that provides batch processing mode for custom CRISPR library design, as well as the most comprehensive tool in sgRNA performance evaluation. By shortening the distance from “silico to bench”, CRISPR-FOCUS facilitates the design of screening experiments and promotes high-throughput functional studies in various scopes.

## Supporting information

S1 File The schema for sgRNA ranking and selection. (DOCX)

S2 File Additional supporting information. (DOCX)
