# Supplementary material for: CRISPR-FOCUS: A web server for designing focused CRISPR screening experiments
Source: PLoS One. 2017 Sep 5;12(9):e0184281. doi: 10.1371/journal.pone.0184281 (PMC5584922; doi:10.1371/journal.pone.0184281)
Supplement: S1 File — (DOCX) [file pone.0184281.s001.docx]

Suppose user requires N sgRNAs for a target gene.

Filter stage:

1. Select all 19 or 20bp sequences upstream of the “NGG” PAM motif, in the coding regions of the target gene;
2. Remove the sequences that:
3. hit SNP / mutant loci;
4. with > 40% of G;
5. with off-target perfect match in the genome;
6. Rank the remaining sequences in descending order of **summary score**, while:

**summary score** = w1 * efficiency score + w2 * specificity score + w3 * conservation score + w4 * commonality score

All weights are determined dynamically by CRITIC method.

1. If the number of remaining sequences is smaller than N, go to Rescue stage, otherwise select the top N sequences with highest summary score to be sgRNA candidates.

Rescue stage:

1. Select all remaining sequences in the Filter stage to be sgRNA candidates.
2. Rescue the sequences with off-target perfect match in **non-exon** regions but not in exon regions.
3. Rank the sequences rescued in 2) in ascending order of the number of off-target perfect matches. If two or more sequences has the same number of off-target matches, rank them in descending order of summary score.
4. Add the rescued sequences in 2) to the sgRNA candidate list in order of the ranks in 3), until the list has a size of N, or all the rescued sequences are added. If the size of candidate list reaches N, exit the Rescue stage.
5. Rescue the sequences with off-target perfect match in **non-coding** regions but not in coding regions;
6. Rank the sequences rescued in 5) in ascending order of the number of off-target perfect matches. If two or more sequences has the same number of off-target matches, rank them in descending order of summary score.
7. Add the rescued sequences in 5) to the sgRNA candidate list in order of the ranks in 6), until the list has a size of N, or all the rescued sequences are added. If the size of candidate list reaches N, exit the Rescue stage.
8. Rescue the sequences with off-target perfect match in **coding** regions;
9. Rank the sequences rescued in 8) in ascending order of the number of off-target perfect matches. If two or more sequences has the same number of off-target matches, rank them in descending order of summary score.
10. Add the rescued sequences in 8) to the sgRNA candidate list in order of the ranks in 9), until the list has a size of N, or all the rescued sequences are added. In both cases, exit the rescue stage.
