# Supplementary material for: CRISPR-FOCUS: A web server for designing focused CRISPR screening experiments
Source: PLoS One. 2017 Sep 5;12(9):e0184281. doi: 10.1371/journal.pone.0184281 (PMC5584922; doi:10.1371/journal.pone.0184281)
Supplement: S2 File — (DOCX) [file pone.0184281.s002.docx]

**Table A. Illustration of CRISPR-FOCUS input/output.**

| **Input** | **Interpretation** |
| --- | --- |
| Organism | Human or Mouse |
| Gene list | Target gene list for library design, either official gene symbol or RefSeq ID, maximum 1000 |
| Number of sgRNA for each gene | Maximum 30 |
| Spacer length | Length of the spacer sequence, 19nt or 20nt |
| Also design control sgRNAs | If chosen, the tool will append positive/negative control sgRNAs to the result, whose number is 10% of total sgRNAs (minimum 10, maximum 500). |
| Avoid cancer recurrent mutation sites | If chosen, CRISPR-FOCUS will filter out sgRNA candidates which overlap with cancer recurrent mutation sites (for Human only). |
| Add promoter/scaffold sequences | If chosen, CRISPR-FOCUS will automatically attach U6 promoter/spCas9 scaffold sequences to the output sgRNA. |

| **Output** | **Interpretation** |
| --- | --- |
| sgID | sgRNA ID, in ‘genename_sgrna#’ format |
| seq | Sequence of this sgRNA |
| chrom | Chromosome ID |
| start | sgRNA start coordinate on genome (1-based, hg38 for human or mm10 for mouse) |
| end | sgRNA end coordinate on genome (1-based, hg38 for human or mm10 for mouse) |
| strand | Strand on which sgRNA resides |
| efficiency | Efficiency score |
| conservation | Average phastCon conservation score for target sequence |
| specificity | Specificity score |
| offhit_nonexon | Number of off-target hits on non-exon regions |
| offhit_noncode | Number of off-target hits on non-coding regions |
| offhit_code | Number of off-target hits on coding regions |

**Table B. Public domain databases that were referenced in sgRNA annotation of CRISPR-FOCUS.**

|  | ***Homo sapiens*** | ***Mus musculus*** |
| --- | --- | --- |
| **Genome assembly** | hg38 | mm10 |
| **SNP database** | dbSNP 147 | dbSNP 142 |
| **Conservation database** | phastCons100way | phastCons60Way |
